# Hemifacial Spasms with Unusual Neurovascular Compression Type: Arterial Cisternal Segment Offender

**DOI:** 10.3390/life16010166

**Published:** 2026-01-19

**Authors:** Hyun Seok Lee, Soung Wook Park, Sang-Ku Park, Kwan Park

**Affiliations:** 1 Department of Neurosurgery, Konkuk University Medical Center, Seoul 05030, Republic of Korea; 20220205@kuh.ac.kr (H.S.L.); 20230333@kuh.ac.kr (S.W.P.); 20200476@kuh.ac.kr (S.-K.P.); 2 Professor Emeritus, Department of Neurosurgery, Sungkyunkwan University School of Medicine, Seoul 06351, Republic of Korea

**Keywords:** hemifacial spasm, microvascular decompression, cisternal segment, interposition technique

## Abstract

(1) Background: Hemifacial spasm (HFS) is most commonly caused by neurovascular compression at the root exit zone (REZ) of the facial nerve; however, isolated compression along the distal cisternal segment is uncommon and remains poorly characterized. This study aimed to analyze the clinical features, intraoperative neurophysiological patterns, and surgical outcomes of patients with HFS caused by cisternal segment arterial compression. (2) Methods: Among 874 patients who underwent microvascular decompression (MVD) for HFS, 18 (2.1%) were identified as having isolated neurovascular conflict at the cisternal segment, all involving the anterior inferior cerebellar artery (AICA). Clinical characteristics, offender location, intraoperative monitoring results including lateral spread response (LSR), brainstem auditory evoked potentials, and postoperative outcomes were retrospectively evaluated. A standardized Teflon interposition technique was used in all cases. (3) Results: Postoperatively, 83.3% of patients experienced immediate spasm relief, and at the latest available follow-up, 94.4% achieved significant improvement without severe complications. (4) Conclusions: Although rare, cisternal segment arterial compression can produce typical HFS and should be considered when REZ compression is unclear or when intraoperative neuromonitoring does not respond as expected. Microvascular decompression using Teflon interposition is a safe and effective treatment option for this anatomically challenging offender location.

## 1. Introduction

Clinically, hemifacial spasm (HFS) manifests as intermittent, involuntary twitching that usually originates in the orbicularis oculi and progressively spreads to the remaining ipsilateral facial musculature. Consequently, patients exhibit facial asymmetry due to the hypercontraction of muscles on the involved side.

Treatment options for HFS encompass a range of conservative strategies, including medications such as muscle relaxants and anticonvulsants, which may provide partial symptom relief. In addition, botulinum toxin injections are widely employed and are considered the standard nonsurgical management because they effectively reduce muscle contractions, although their benefits are temporary and require repeated administration. Despite these modalities, microvascular decompression (MVD) remains the most reliable, definitive, and durable treatment option, as it directly addresses the underlying neurovascular conflict. By eliminating the neurovascular conflict of the facial nerve at the root exit zone, MVD offers the unique advantage of completely resolving symptoms and restoring normal facial function in the majority of patients [1,2,3,4,5,6,7,8,9].

The pathogenesis of HFS is thought to arise from vascular compression of the facial nerve at its root exit zone (REZ) near the brainstem, which induces focal demyelination and aberrant ephaptic transmission between nerve fibers. And the overall rate of achieving a spasm-free state following MVD in patients with HFS is estimated to be around 90%. However, approximately 10% of patients may suffer from residual spasms, encountering delayed recurrence [2,4,10,11].

In HFS, proximal cisternal offenders near the REZ are occasionally reported, but there is no systematic compilation or reporting on those located in the distal cisternal segment at the entrance to the IAC. The aim of this study is to document this occurrence.

## 2. Materials and Methods

### 2.1. Patient Cohort

This study was designed as a retrospective observational case series. We retrospectively analyzed the medical records of 874 patients who underwent MVD for HFS at Konkuk University Medical Center between September 2020 and July 2025. All MVD surgeries were performed with brainstem auditory evoked potential (BAEP), facial nerve motor evoked potential (facial MEP), and lateral spread response (LSR, same as abnormal muscle response, AMR) monitoring by an experienced neurophysiologist. All 874 patients’ characteristics and clinical information are summarized in Table 1.

**Table 1:** Clinical characteristics of all 874 patients who underwent MVD surgery with HFS.

Clinical Characteristics	
Median age at surgery (range, years)	57 (19–82)
Sex (Male/Female)	239:635
Lesion side (Left/Right)	449:425
Postoperative to discharge (days, range)	5.35 (3–20) *
**Compression type**	
Arachnoid type	157 (18.0%)
Loop type	134 (15.3%)
Branch type	52 (5.9%)
Tandem type	198 (22.7%)
Sandwich type	77 (8.8%)
Perforator type	150 (17.2%)
Encircling type	53 (6.1%)
Arterial cisternal segment offender	18 (2.1%)
Vein offender	9 (1.0%)
Miscellaneous or uncategorized	26 (3.0%)

**Figure 1:**
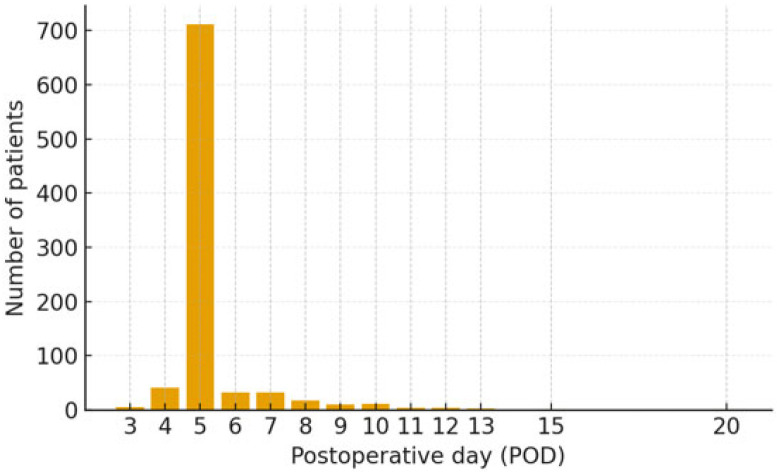
Distribution of patients by postoperative to discharge days.

MVD, microvascular decompression; HFS, hemifacial spasm; * Postoperative to discharge days distribution is shown in Figure 1.

Among this cohort of 874 patients, 18 (2.1%) were found to have cisternal segment offenders. Cisternal segment offenders were identified based on intraoperative microscopic findings and neuromonitoring responses. A cisternal segment offender was confirmed when intraoperative inspection revealed no definite vascular compression at the REZ or when REZ compression was ambiguous and intraoperative neuromonitoring (IOM) showed no Sang-Ku sign (SKS) during decompression, with the lateral spread response (LSR) remaining unchanged or not disappearing after an observation period following decompression. All assessments were performed by a single experienced neurosurgeon and a single experienced neurophysiologist, ensuring consistency in evaluation across cases. These 18 patients included 5 males and 13 females, with 13 having right-sided lesions and 5 having left-sided lesions. The patients’ median age was 54.5 years (age range 37–67 years), and the median duration of symptoms was 39 months (range, 18–228 months). Of the 18 patients, 11 (61.1%) underwent botulinum toxin injection treatment prior to MVD surgery. In addition, two of the patients had a history of hypertension and were taking medication for it; the other sixteen patients had no prior medical history. [Table 2]

### 2.2. Surgical Procedures and Intraoperative Neuromonitoring

The MVD procedures were performed through a retromastoid suboccipital craniotomy (RMSOC) with the park bench lateral position. Intraoperative neuromonitoring was routinely applied in all cases and included the lateral spread response (LSR), also known as the abnormal muscle response (AMR); facial motor evoked potentials (fMEPs); and brainstem auditory evoked potentials (BAEPs) with the real-time monitoring method [12]. The intraoperative changes in LSR were classified as follows: no LSR observed at all during surgery; disappearance before decompression after dural incision; complete disappearance immediately after decompression; delayed disappearance after decompression; no disappearance after decompression. And all patients underwent decompression using Teflon felt pledgets (Bard Peripheral Vascular, Inc., Tempe, AZ, USA).

### 2.3. Perioperative Assessment

All patients underwent preoperative hearing examination, including pure-tone audiometry (PTA) and speech audiometry (SA). Patients were also evaluated for the presence of preoperative lateral spread response (LSR) by an experienced neurophysiologist. A brain computed tomography scan (CT) was performed immediately after surgery and prior to discharge, and a same hearing examination was conducted before discharge. The postoperative outpatient follow-up visit occurred one month later, at which time the postoperative LSR was evaluated.

### 2.4. Statistical Analysis

Given the small sample size, this study was designed as a descriptive exploratory analysis, and only descriptive statistics were used.

## 3. Results

In all 18 patients, the offending vessel was the anterior inferior cerebellar artery (AICA), and the decompression procedure for all patients was an interposition technique, which placed Teflon felt between the facial nerve and the compression AICA.

**Figure 2:**
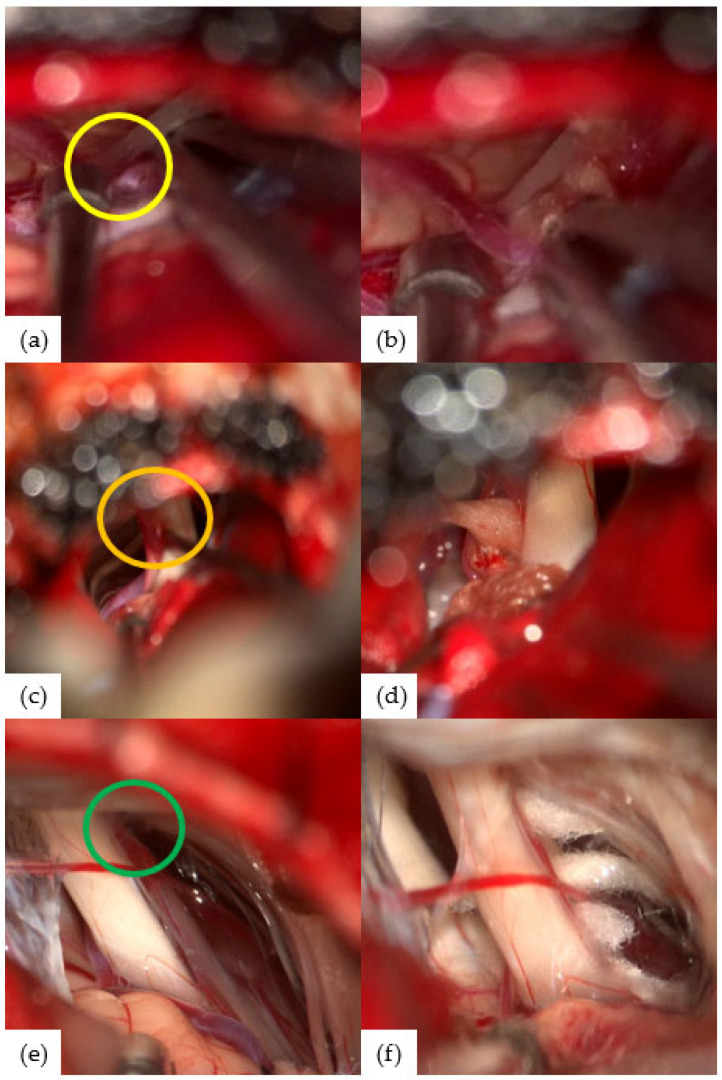
(**a**) Microscopic surgical finding of case 13 with left HFS. Neurovascular compression site was proximal 1/3 (yellow circle). (**b**) After decompression of case 13 patient with interposition technique. (**c**) Microscopic surgical finding of case 11 with left HFS. Neurovascular compression site was middle 1/3 (orange circle). (**d**) After decompression of case 11 patient with interposition technique. (**e**) Microscopic surgical finding of case 18 with right HFS. Neurovascular compression site was distal 1/3 (green circle). (**f**) After decompression of case 18 patient with interposition technique.

To evaluate the compression site within the cisternal segment of the facial nerve, it was divided into three equal thirds: proximal, middle, and distal. The proximal third near the brainstem was present in five patients (Figure 2a,b), the middle third in six patients (Figure 2c,d), and the distal third near the IAC entrance in five patients (Figure 2e,f); in two patients, it could not be clearly determined.

In five patients (27.8%), LSR was not observed intraoperatively. In 12 patients (66.7%), LSR disappeared after immediate decompression. One patient (5.6%) showed delayed disappearance of LSR, during the closure of dura.

In this series, postoperative spasm outcome was assessed immediately after surgery (within 3 days) and at the latest available follow-up; 14 patients had a follow-up of 6 months or longer, based on the patient’s subjective estimation of residual spasm severity relative to the preoperative condition, with “20% residual spasm” indicating that patients perceived that approximately 20% of their preoperative symptoms persisted. The follow-up duration ranged from 24 to 1469 days, with a median follow-up of 245 days (interquartile range, 211–648 days).

Short-term outcomes showed immediate complete resolution of spasm in 15 of 18 patients. In the remaining three patients, minimal residual spasm (approximately 20% of preoperative symptoms) was observed immediately after surgery; at 6 months, two patients continued to report minimal residual spasm, whereas one patient showed delayed complete resolution. Moreover, 14 of the 18 patients had follow-up durations of at least 6 months. Among the three patients who showed about 20% residual spasm immediately after surgery compared to preoperative conditions, two remained unchanged during the follow-up period, while one showed a delayed response and the spasm resolved. Additionally, spasm recurred in two patients who had no spasm immediately after surgery: one experienced a mild recurrence of approximately 20% compared to preoperative symptoms, while the other showed a complete recurrence of the same state as before surgery.

BAEP changes were observed in four patients during surgery. In two patients, the amplitude decreased by 100% but fully recovered by the end of surgery. Of the other two patients, one patient experienced an decrease in BAEP amplitude to 30% of baseline twice, while the other patient’s amplitude decreased to half of baseline, fully recovering by the end of surgery.

Facial palsy was observed in one patient (5.6%) immediately after surgery, with House–Brackmann (H-B) grade III, which improved to H-B II approximately 10 days later. Additionally, delayed facial palsy occurred in two patients (11.1%), with separate grades of H-B II and III.

Postoperative LSR examinations were performed in the same laboratory as preoperative examinations during outpatient visits approximately one month after surgery. Preoperative LSR was detected in 17 out of 18 patients, and among these 17 patients, postoperative LSR was not detected at the first outpatient visit in 13 patients. Four patients showed LSR during postoperative examination. Of these, two had no spasm at all after surgery and were not retested. In one patient LSR persisted in tests, twice. Although spasm was absent immediately after surgery, it developed in approximately 20% over time.

And one patient had spasm at about 20%, and after approximately one year of outpatient follow-up observation, the LSR had disappeared and the spasm also resolved. One patient who did not show LSR before surgery also did not show it after surgery; this patient is one of three who retained approximately 20% spasm postoperatively. And one patient who did not show postoperative spasm did not undergo postoperative LSR examination.

We have published the Sang-Ku sign (SKS), a specific electromyography (EMG) pattern which is helpful for identifying the site of compression [13], and have identified this as it is actually being used clinically in our MVD surgeries. As a result, SKS was observed in 14 patients, while it was not possible to determine this in 4 patients because they underwent surgery before SKS was clinically utilized in our institution. 

The intraoperative findings and prognosis of cisternal offender cases is summarized in Table 3.

## 4. Discussion

The modern MVD technique was first introduced by Jannetta in 1977, and was later widely disseminated through multiple publications that demonstrated its potential as a curative procedure for HFS [14,15,16,17,18]. Since then, microsurgical techniques have undergone continuous improvement, and the recent literature reports cure rates for hemifacial spasm of approximately 85–96% [4,10,19,20,21,22]. Although our study was relatively small with a cohort of 18 patients, 14 had follow-up durations of at least 6 months. Of these, 10 patients remained spasm-free during follow-up, whereas 4 patients experienced residual or recurrent spasm. Two patients continued to report minimal residual spasm (approximately 20% of preoperative symptoms), one patient developed partial recurrence (approximately 20% of preoperative symptoms) after an initially spasm-free period, and one patient experienced recurrence to a severity comparable to the preoperative state. In contrast, one patient who had minimal residual spasm immediately after surgery showed complete resolution during long-term follow-up. Including patients with minimal residual spasm (approximately 20% of preoperative symptoms), effective outcomes were observed in 17 patients (94.4%), and among patients with follow-up of at least 6 months, effective outcomes were observed in 13 of 14 patients.

As is widely known, the most common vessel causing hemifacial spasm is the AICA, followed by the posterior inferior cerebellar artery (PICA) [2,19,23,24,25]. The PICA mostly originates from the VA, and ascends from the medulla toward the pons, typically forming a loop at or below the pontomedullary junction and descending. Therefore, when the PICA is the cause of hemifacial spasm, it typically makes a groove slightly below the REZ, forms a loop, and compresses the facial nerve. So, it is difficult for the PICA to compress the cisternal segment of the facial nerve, which emerges from the brainstem near the pontomedullary junction and heads toward the internal auditory canal [25]. Taking these aspects into consideration, all 18 cisternal offenders in this study are thought to have been AICA offenders. Generally, the AICA commonly forms a meatal loop near the internal auditory canal and often contact with the cisternal segment of the seventh or eighth cranial nerve. If there is an AICA-PICA common trunk or significant vascular variation, other offenders are possible but considered rare. Furthermore, while veins attached to the brainstem are visible in this location, veins traversing the arachnoid space are uncommon. This also suggests that venous offenders are rare in the cisternal segment.

The lateral spread response (LSR), frequently referred to as the abnormal muscle response (AMR), is a pathognomonic electromyographic finding characteristic of hemifacial spasm (HFS). In a normal facial nerve, electrical stimulation of a specific nerve branch results in the contraction of only the muscles innervated by that branch. However, in patients with HFS, stimulation of one branch elicits a delayed depolarization in muscles innervated by a different branch. This phenomenon indicates a pathological “cross-talk” between adjacent nerve fibers. During MVD surgery, LSR serves as a valuable and objective intraoperative marker for evaluating the adequacy of neurovascular decompression. Immediate intraoperative disappearance of the LSR following mobilization of the offending vessel is widely regarded as a strong predictor of favorable postoperative outcomes. However, this LSR is not measured during surgery in all patients. Therefore, we identified and published another useful finding: the Sang-Ku sign (SKS) [13]. There should be no doubt that when there is no offender in the REZ, one should obviously check for a cisternal segment of the facial nerve. And even when vessels are visible around the REZ but compression is not clear, or when there is no change in neuromonitoring signs (i.e., LSR or SKS) even after decompressing a vessel thought to be the offender at the REZ, it is still clearly necessary to check the whole segment of the facial nerve. Among the 13 patients who presented with LSR during surgery, 12 (92.3%) experienced immediate disappearance of LSR following decompression of neurovascular conflict in the cisternal segment, supporting this idea. Additionally, the “SKS” sign which was published recently was observed in all patients during decompression except for the four who underwent surgery before this sign was established. Notably, it was observed in all five patients who did not show LSR, suggesting it sufficiently fulfilled its original purpose as an additional marker to supplement LSR. Checking the location of cisternal segment offenders, we can see they were evenly distributed across the proximal, middle, and distal segments. Generally, the distal cisternal segment of the facial nerve is myelinated, so even with vascular contact, HFS rarely occurs. However, in certain rare cases like our study’s patients, it is possible to identify the cause of HFS. This is thought to be due to individual anatomical differences in the facial nerve or variations in nerve sensitivity, and further study is needed to determine the cause. But since it is not possible to perform procedures such as facial nerve biopsy, there are limitations to assessing the individual causes for each patient.

In this study, recurrence was observed in one patient with case 4. Immediately after surgery, she experienced almost no spasm. However, shortly after discharge, she developed minimal spasm, which progressively worsened over time. Approximately three years later, she reported severe spasm similar to that experienced before surgery. The patient had contact between the PICA and the pontomedullary junction, but there was no indentation, and there was no change in the LSR after decompression of the PICA. Therefore, the cisternal segment of the facial nerve was checked, and a branch of the AICA was found to be in contact on the lateral side of the middle third. This was decompressed using a Teflon interposition technique, and immediately after Teflon insertion, the SKS was visualized and the LSR disappeared. The most common cause of recurrence is the offender being missed [3,26]. There is also a possibility that another third culprit was involved. Also the cause of recurrent spasm in this patient seemed to be incomplete decompression rather than a temporary recurrence of symptom, considering that the symptom gradually began to worsen after discharge, and also that several years had passed and the condition was persistent. Additionally, it is possible that the course of the offending vessel was altered by the interposition technique, leading to new neurovascular conflicts. Reevaluation is being considered.

Something we should consider here is the surgical technique of transposition versus interposition, which has been a recurring topic in numerous studies, even including a systemic review of over 15,000 patients, which was published recently [27]. Historically, interposition has been the standard technique for MVD. This method involves placing a prosthesis, typically shredded Teflon felt, at the site of neurovascular conflict. The theoretical basis relies on creating a physical barrier that absorbs the pulsatile pressure of the vessel [14,16,18]. However, this technique carries inherent risks, including the potential for the prosthesis to slip or induce a foreign body reaction, known as a Teflon granuloma [28]. Therefore, there are reports indicating that transposition surgery yields better outcomes [27,29]. The transposition technique aims to permanently mobilize the offending artery away from the facial nerve. This is achieved by mobilizing the vessel and then securing it to the dura or adjacent structures using a Teflon sling or fibrin glue. Theoretically, transposition is considered to be a superior method because it completely removes the compression vector and minimizes the amount of foreign material in direct contact with the REZ, thereby reducing the risk of granuloma formation. However, transposition is not achievable in all HFS patients; a notable instance is the perforator type, where numerous perforators supply the brainstem [11,30,31]. Similarly, most cisternal segment offenders in our study are difficult to transpose. In our study, cisternal offender cases generally could not undergo transposition because the eighth cranial nerve is anterior to the surgical field, making it impossible to transpose. Therefore, all cases in our study underwent decompression via interposition. Considering the prognosis for spasm in this study, it seems reasonable to perform decompression using the interposition technique for cisternal offenders rather than forcing transposition, which carries risks such as vestibulocochlear nerve injury due to stretching damage or vascular damage. Further study with a larger number of cases and on the long-term prognosis for study would likely provide additional evidence.

This study has several limitations. Because this study was intended as a descriptive exploratory analysis with a small sample size, no formal statistical hypothesis testing or comparative statistical analysis was performed, and the results should be interpreted descriptively rather than inferentially. In addition, despite being performed by experienced personnel, identification of cisternal segment offenders relied on intraoperative judgment and neuromonitoring responses, which may introduce observer bias. Furthermore, the wide range of follow-up durations limits definitive conclusions regarding long-term prognosis, particularly with regards to recurrence rates.

## 5. Conclusions

Hemifacial spasm may be caused by neurovascular compression along the cisternal segment of the facial nerve, even beyond the root exit zone. When the REZ compression or intraoperative neuromonitoring findings are inconclusive, inspection of the entire facial nerve with interposition decompression provides favorable clinical outcomes with acceptable complication rates.

## Figures and Tables

**Table 2:** Clinical information of 18 patients with cisternal offenders.

Clinical Characteristics							
Median age at surgery (range, years)					54.5 (37–67)		
Sex (M/F)					5:13		
Lesion side (L/R)					5:13		
Symptom duration, months: median (range)					39 (18–228)		
Preoperative LSR response					17 (94.4%)		
**No.**	**Sex**	**Age**	**Spasm Side**	**Preoperative Treatment**	**Symptom Duration (Month)**	**Preoperative** **LSR**	**Co-Morbidity**
1	M	57	R	None	24	Positive	None
2	M	45	R	None	30	Positive	None
3	F	53	R	None	36	Positive	None
4	F	61	R	BTX	84	Positive	None
5	F	49	R	None	18	Positive	None
6	F	56	R	BTX	36	Positive	None
7	F	44	R	BTX	31	Positive	None
8	F	66	R	BTX	228	Positive	None
9	M	37	R	BTX	60	Positive	None
10	F	52	L	BTX	144	Positive	None
11	M	54	L	None	60	Positive	None
12	F	46	R	BTX	40	Positive	None
13	M	57	L	None	24	Positive	None
14	F	59	R	BTX	120	Positive	None
15	F	56	L	BTX	66	Positive	None
16	F	54	L	BTX	38	None	HTN
17	F	67	R	BTX	66	Positive	None
18	F	61	R	None	36	Positive	HTN

M, male; F, female; R, right; L, left; No., number; LSR, lateral spread response; BTX, botulinum toxin; HTN, hypertension.

**Table 3:** Surgical findings and prognosis of 18 patients with cisternal offenders.

Case No.	Offending Site	Intra-Op. LSR	SKS	Intra-Op. BAEP	Postop. Spasm		Postop. LSR	Postop. Facial Palsy	F/U (Days)
					~3D	Latest F/U			
1	Proximal 1/3	Disappeared after decompression	N/A	No change	0	0	Disappeared	None	1469
2	Proximal 1/3	Disappeared after decompression	N/A	100% loss, full recovery	0	0	Disappeared	None	137
3	Proximal 1/3	Disappeared after decompression	N/A	No change	0	0	Disappeared	None	1056
4	Middle 1/3	Disappeared after decompression	N/A	No change	0	100%	Disappeared, but no test was performed after recurrence	None	1014
5	Middle 1/3	Disappeared after decompression	+	No change	0	0	Disappeared	Delayed H-B III	711
6	Not mentioned	Disappeared after decompression	+	70% loss twice, full recovery	0	0	Disappeared	Immediate H-B III	505
7	Not mentioned	No intraoperative LSR	+	No change	0	0	Disappeared	Delayed H-B II	648
8	Distal 1/3	Disappeared slowly	+	No change	0	20%	Showed twice	None	384
9	Middle 1/3	Disappeared after decompression	+	No change	0	0	Disappeared	None	43
10	Middle 1/3	No intraoperative LSR	+	100% loss, full recovery	20%	0	Disappeared at 2nd test	None	400
11	Middle 1/3	No intraoperative LSR	+	No change	20%	20%	Disappeared	None	220
12	Middle 1/3	Disappeared after decompression	+	No change	0	0	Disappeared	None	223
13	Proximal 1/3	Disappeared after decompression	+	No change	0	0	Disappeared	None	230
14	Proximal 1/3	No intraoperative LSR	+	No change	0	0	Disappeared	None	223
15	Distal 1/3	Disappeared after decompression	+	No change	0	0	Showed	None	211
16	Distal 1/3	No intraoperative LSR	+	No change	20%	20%	Disappeared	None	260
17	Distal 1/3	Disappeared after decompression	+	No change	0	0	No examination	None	24
18	Distal 1/3	Disappeared after decompression	+	50% loss, full recovery	0	0	Showed	None	101

No., number; LSR, lateral spread response; SKS, Sang-Ku sign; BAEP, brainstem auditory evoked potential; D, days; F/U, follow-up; H-B, House–Brackmann grade; N/A; not available.

## Data Availability

The data presented in this study are available on request from the corresponding author.
